# Reliability and validity of the Thai pediatric quality of life inventory™ 3.0 neuromuscular module

**DOI:** 10.1186/s12955-020-01492-z

**Published:** 2020-07-20

**Authors:** Apirada Thongsing, Yanin Suksangkarn, Oranee Sanmaneechai

**Affiliations:** Division of Neurology, Department of Pediatrics, Faculty of Medicine Siriraj Hospital, Mahidol University, Wanglang Road, Bangkoknoi, Bangkok, 10700 Thailand

**Keywords:** Reliability, Validity, Thai pediatric quality of life inventory™ 3.0 neuromuscular module, PedsQL™ 3.0 NMM Thai version

## Abstract

**Background:**

Neuromuscular disorders (NMDs) are chronic illnesses that adversely impact the lives of patients and their families. The Pediatric Quality of Life™ 3.0 Neuromuscular Module (PedsQL™ 3.0 NMM) was designed to assess health-related quality of life (HRQoL) among children with NMDs. The objective of this cross-sectional study is to evaluate the reliability and validity of the PedsQL™ 3.0 NMM Thai version.

**Methods:**

Formal permission to translate the PedsQL™ 3.0 NMM into Thai language was granted by the inventor, and the translation process followed linguistic translation guidelines. The PedsQL™ 3.0 NMM Thai version was administered to children with NMD and their parents/caregivers at the Division of Neurology, Department of Pediatrics, Faculty of Medicine Siriraj Hospital, Mahidol University, Bangkok, Thailand. Re-test was performed within 2–4 weeks after the initial test.

**Results:**

One hundred and three children with NMD and their parents or caregivers were enrolled. Internal reliability as measured by Cronbach’s alpha was > 0.7 (total score: child α = 0.88, parent α = 0.91). Test-retest reliability showed good agreement with an intraclass correlation coefficient (ICC) of 0.69 and 0.82 for the total score of the child report and the parent report, respectively. The mean (SD) quality of life total score for the child self-report was 74.9 (13.9) among ambulatory patients, and 60.7 (15.2) among non-ambulatory patients (maximum quality of life score is 100). The mean total quality of life score for the parent proxy-report was 70 (14.5) among ambulatory patients, and 55.2 (18.3) among non-ambulatory patients. The child total score was in good agreement with the parent/caregiver total score.

**Conclusions:**

PedsQL™ 3.0 NMM Thai version is a reliable and valid measure of HRQoL in Thai children with NMDs.

## Background

Neuromuscular disorders (NMDs) are diseases that originate from pathologic changes in anterior horn cells (AHCs), peripheral nerves, neuromuscular junctions (NMJs), and/or muscles. The prevalence of NMDs has been estimated to range from 1 to 10 per 100,000 population [1]. NMDs are chronic illnesses that manifest via motor deficits that impair the physical, family, social, and school-related functioning of patients and their families. Health-related quality of life (HRQoL) is an important outcome indicator for disease progression evaluation, clinical trials, and research in pediatric populations with chronic health conditions [2]. There are several HRQoL questionnaires, including both generic and disease-specific versions [3].

The Pediatric Quality of Life (PedsQL™) 4.0 Generic Core Scales questionnaire yields information on the physical, emotional, social, and school functioning of the child during the previous 4 weeks [4]. Generic HRQoL measurements are important for assessing outcomes between different populations and interventions, whereas disease-specific HRQoL measurements have enhanced sensitivity for evaluating health domains and concerns relating to specific chronic health conditions. The PedsQL™ 3.0 Neuromuscular Module was designed to assess the quality of life of children with NMD aged 2–18 years [5].

Both generic and disease-specific measures should be evaluated in pediatric patients with chronic diseases to achieve a thorough assessment of HRQoL [6–8]. A Thai language version of the PedsQL™ 4.0 Generic Core Scale is available for the general population [9], but a validated Thai language version of the PedsQL™ 3.0 NMM is not yet available. Thus translation of PedsQL 3.0 NMM is crucial to evaluate this population.

The primary objective of this study was to evaluate the reliability and validity of the Thai language translation of the PedsQL™ 3.0 NMM in Thai children with NMD aged 2–18 years. The secondary objective is to evaluate quality of life in Thai children with NMD through PedsQL™ 3.0 NMM. The Thai versions of this questionnaire will be used in both clinical settings (for example to assess the efficacy of treatments such as gene therapy), and in public health, informing national health policy in pediatric NMD towards improving quality of life for children with NMDs.

## Methods

### Study design and patient population

This cross-sectional study was performed during August 2016 to March 2017. Children with NMD were recruited from the Faculty of Medicine Siriraj Hospital, Mahidol University Pediatric Neuromuscular clinic. Inclusion criteria were: 1) neuromuscular diagnosis; confirmed by genetic testing, electromyography/nerve conduction study, or muscle biopsy 2) age 2–18. Children were ineligible if they had a chronic illness in addition to their neuromuscular diagnosis, or limited Thai proficiency. Subject selection was carried out by a researcher approaching all children with NMD who come to neuromuscular clinic and attend the annual Duchenne Muscular Dystrophy meeting. The questionnaire was administered in the pediatric neuromuscular clinic to parents and their child. The subjects filled out the questionnaire independently, without interaction between the parents and their child. The re-test was performed in person in the clinic or by mail. The subject selection was done using purposive sampling. A recruitment pool of all patients in neuromuscular clinic and annual Duchenne day, a total of 103 patients was approached to participate and all agreed to participate. Informed assent was obtained from all study children, and written informed consent was obtained from all study parents or caregivers. The protocol for this study was approved by the Siriraj Institutional Review Board.

### Measures and procedures

#### PedsQL™ 3.0 NMM

The PedsQL™ 3.0 NMM consists of child self-report and parent proxy-report with 25 items in 3 domains, including ‘About My/My Child’s Neuromuscular Disease’ (17 items), ‘Communication’ (3 items), and ‘About Our Family Resources’ (5 items). Child self-reports vary according to age group, as follows: 5–7 years (young children), 8–12 years (children), and 13–18 years (teens). Parent proxy-reports also vary according to age group, as follows: 2–4 years (toddler), 5–7 years (young children), 8–12 years (children), and 13–18 years (teens). Both child self-reports and parent proxy-reports versions are parallel and have the same numbers of items in 3 domains except child self-report for young children (5–7 years) which has only one domain (About my neuromuscular disease). How much of a problem each item has been within the last month is rated using a 5-option scale, with 0 indicating never a problem, and 4 indicating almost always a problem. The self-administered form used for the 5–7 years group uses a 3-point scale consisting of emojis to elicit the level of difficulty (0 with a big smiling emoji = not at all a problem; 2 with an indifferent emoji = sometimes a problem; and, 4 with a sad emoji = almost always a problem). Items are reverse scored and linearly transformed to a 0 to 100 point scale (0 = 100 points; 1 = 75 points; 2 = 50 points; 3 = 25 points; and, 4 = 0 points), with a higher score indicating a better HRQoL. The translation process was performed after receiving formal approval to do so by Dr. James Varni, the inventory and copyright owner of the PedsQL™. Translation of the PedsQL™ 3.0 NMM into Thai was performed according to linguistic translation guidelines using the forward-backward translation method [10]. All steps were completed and the final version was accepted by the MAPI Research Institute in Lyon, France on behalf of Dr. James W. Varni.

The PedsQL™ 3.0 NMM Thai version was administered to NMD children and their parent/caregiver separately at the neuromuscular clinic. For studies that assess children’s QoL through both children’s and parents’ perspectives, it is recommended to separate them. We do not let the parents and children to have interaction because parents may influence children’s answers [11, 12]. If a child was unable to read, a research assistant read the questionnaire verbally and recorded the responses. A research assistant that was not a healthcare provider to the study patients was chosen in order to reduce bias associated with favorable interpretation of patient and caregiver/parent responses. Demographic characteristics and clinical manifestation data were reviewed from medical records. Psychometric properties were established and a re-test was performed within 2–4 weeks after the initial test during a routine clinical visit or by mail.

#### Sample size calculation and statistical analysis

We calculated the sample size by estimating the child-parent agreement ICC of the total score to be 0.5 (expected intraclass correlation) ± 0.15 (the distance from correlation to limit) with 95% CI, the sample size was *n* = 98 (child-parent agreement ICC found in the previous study [13] ranges from 0.279–0.681). Data were analyzed with SPSS (Statistical Package for the Social Sciences) 2.0 version with *p* value set at ≤0.05. The distribution of the demographic data was evaluated using Shapiro-Wilk tests. Demographic data were reported as percentage, median, IQR and range. Feasibility of the questionnaire was assessed by the percentage of missing data (unanswered items) [2, 9, 14, 15] The percentage of scores at the extremes of the scaling range, that is, the maximum possible score (ceiling effect) and the minimum possible score (floor effect) [16]. Surveys with small floor or ceiling effects (no more than 15%) are considered to meet acceptable measurement standards, while surveys with moderate floor or ceiling effects (> 15%) are considered less precise in measuring latent constructs at the extremes of the scale [17]. Scale internal consistency reliability was determined at the first evaluation by calculating Cronbach’s alpha coefficient [18]. Scales with reliabilities ≥0.70 are considered satisfactory. Test-retest reliability for the Thai version scale was assessed for a subset of the sample (*n* = 74) using intraclass correlation coefficients (ICCs) [19]. Intraclass correlations range from − 1 to 1, with higher values indicating better agreement. ICCs ≤0.40 were designated as poor to fair agreement, 0.41–0.60 moderate agreement, 0.61–0.80 good agreement, and 0.81–1.00 excellent agreement [20–22]. Agreement between child self-report and parent proxy-report of the Thai version was determined using ICCs [23], which offers an index of absolute agreement because it takes into account the ratio between subject variability and total variability [24]. We want to assess the agreement between child-report and parent-report to learn about how the parent’s assessment of their child’s quality of life is related to the child’s report [25–27]. Parent’s perception of their child’s HRQL also determined the utilization of health care services [4]. Known-group validity was assessed between ambulatory and non-ambulatory groups using the independent sample t-test to compare the first evaluation scores.

## Results

### Patient characteristics

A total of 103 NMD patients from 94 families were enrolled. Demographic characteristics of the NMD patients are shown in Table 1. The median age of patients at the time of visit was 11 years (IQR 7). The majority (55%) of NMD patients had Duchenne muscular dystrophy (DMD).

**Table 1:** Demographic characteristics of 103 NMD patients

Data	Median ± IQR (range) or n (%)
Age of onset (years)	4 ± 5 (0–11)
Age at time of the evaluation (years)	11 ± 7 (2–18)
2–4	7 (6.8)
5–7	19 (18.4)
8–12	38 (36.9)
13–18	39 (37.9)
Male	78 (75.7)
Diagnosis	
Duchenne Muscular Dystrophy (DMD)	57 (55.3)
Charcot-Marie-Tooth (CMT)	10 (9.7)
Spinal Muscular Atrophy (SMA)	8 (7.8)
Other	28 (27.2)
Non-ambulatory patients	49 (47.6)
Positive Family History	29 (28.2)
Currently in school	52 (50.5)

### Feasibility

The percentage of missing responses for the child self-report at the item level was 3.88%. Four NMD children (age 7, 8, 12, and 17 years) could not complete the child self-report questionnaire due to intellectual disability and mechanical ventilation. The parent-report was completed for these two patients. The percentage of missing data responses for the parent-report was 0.97%.

### Reliability

#### Internal consistency reliability

Scale was determined at the first evaluation by calculating Cronbach’s alpha coefficient. Most child self-report subscales and parent proxy-report subscales exceeded the minimum reliability standard of 0.7 (Table 2). Cronbach’s alpha coefficient for child self-report total score was 0.88 (subscales range 0.54–0.89) and for parent proxy-report total score was 0.91 (subscales range 0.81–0.92). Ceiling effect was observed in both the child self-report and parent proxy-report in the communication subscale.

**Table 2:** Internal consistency of the Thai version of PedsQL™ 3.0 NMM

Scale (# items)	n	Cronbach’s alpha	Mean	SD	%Floor	%Ceiling
Child self-report						
Total [25]	91	0.88	67.9	16.1	2.9	1.1
About My Neuromuscular Disease [17]	91	0.89	65.1	19.6	1.1	1.1
Communication [3]	74	0.82	69.6	23.6	9.5	20.3
About Our Family Resources [5]	74	0.54	74.3	15.3	1.4	4.1
Parent Proxy-report						
Total [25]	103	0.91	72.8	11.0	1.0	1.0
About My Child’s Neuromuscular Disease [17]	103	0.89	72.7	12.2	1.0	1.0
Communication [3]	103	0.92	77.0	23.8	4.9	29.1
About Our Family Resources [5]	103	0.81	70.8	15.8	1.0	1.0

#### Test-retest reliability

A subset of children (*n* = 64) and parents (*n* = 74) completed the PedsQL™ 3.0 NMM a second time 2–4 weeks after taking the initial test during a routinely scheduled clinic visit or by mail (Table 3). ICCs of test-retest reliability showed good agreement in some subscales for the child self-report and good to excellent agreement for all subscales for the parent proxy-report.

**Table 3:** Test-retest reliability of the Thai version of PedsQL™ 3.0 NMM

Scale (# items)	Intraclass correlation coefficient, ICC (95% CI)
Child self-report	
Total [25]	0.69 (0.54, 0.80)
About My Neuromuscular Disease [17]	0.76 (0.63, 0.85)
Communication [3]	0.35 (0.10, 0.56)
About Our Family Resources [5]	0.40 (0.08, 0.63)
Parent proxy-report	
Total [25]	0.82 (0.72, 0.89)
About My Child’s Neuromuscular Disease [17]	0.84 (0.74, 0.90)
Communication [3]	0.63 (0.48, 0.75)
About Our Family Resources [5]	0.74 (0.62, 0.83)

*ICC* intraclass correlation, ICCs are designated as ≤0.40 poor to fair agreement, 0.41–0.60 moderate agreement, 0.61–0.80 good agreement, and 0.81–1.00 excellent agreement

#### Parent-child agreement

Agreement between child self-report and parent proxy-report of the Thai version scale was determined using ICCs. There was moderate agreement in all subscales (Table 4).

**Table 4:** Parent-Child Agreement of the Thai version of PedsQL™ 3.0 NMM

Scale (# items)	Intraclass correlation coefficient, ICC (95% CI)
Total [18]	0.71 (0.57, 0.80)
About My/My Child’s Neuromuscular Disease [17]	0.75 (0.65, 0.83)
Communication [3]	0.24 (0.01, 0.45)
About Our Family Resources [5]	0.33 (0.07, 0.54)

*ICC* intraclass correlation, ICCs are designated as ≤0.40 poor to fair agreement, 0.41–0.60 moderate agreement, 0.61–0.80 good agreement, and 0.81–1.00 excellent agreement

#### Known-group validity

Known-group validity was assessed between ambulatory and non-ambulatory groups using the independent samples *t*-test to compare the first evaluation score (Table 5). The mean of all domains was higher in the ambulatory group than in the non-ambulatory group. The scales ‘About My Neuromuscular Disease’ and ‘Total score’ were significantly higher in the ambulatory group in the child self-report. The ‘Total score’, ‘About My Child’s Neuromuscular Disease’, ‘About Our Family Resources’ and ‘Communication’ scales were significantly higher in the ambulatory group in the parent proxy-report.

**Table 5:** Construct validity of the Thai version of PedsQL™ 3.0 NMM, comparing ambulatory and non-ambulatory

Scale (# items)	Ambulatory		Non-ambulatory		P	Difference (95% CI)
	Mean	SD	Mean	SD		
Child self-report	(*n* = 46)		(*n* = 45)			
Total [25]	74.9	13.9	60.7	15.2	< 0.001	14.2 (8.1, 20.3)
About my neuromuscular disease [17]	73.7	15.1	56.3	19.8	< 0.001	17.4 (10.1, 24.7)
Communication [3]	70.3	22.3	69.1	24.9	0.82	1.2 (−9.9, 12.4)
About our family resources [5]	78.1	16.0	71.3	14.2	0.06	6.8 (−0.2, 13.8)
Parent proxy-report	(*n* = 54)		(*n* = 49)			
Total [25]	70.0	14.5	55.2	18.3	< 0.001	14.8 (8.4, 21.3)
About my child’s neuromuscular disease [17]	69.8	14.8	53.8	20.1	< 0.001	16.0 (9.2, 22.9)
Communication [3]	75.0	28.3	61.6	31.6	0.02	13.4 (1.7, 25.1)
About Our Family Resources [5]	67.9	21.0	56.0	23.5	0.01	11.9 (3.2, 20.6)

## Discussion

This study confirms that the PedsQL™ 3.0 NMM Thai version is a valid and reliable instrument for evaluating quality of life in the pediatric NMD population. There were only a few missing item responses, which indicated that children and their parents were able to provide good quality data. The missing item responses in the self-report were due to the severe physical and intellectual limitations of a few children. The missing responses in the parent proxy-report could be due to misunderstanding of the provided instructions. No floor effect was observed for any of the subscales, but a ceiling effect was observed in the communication subscale of the parent proxy-report. The overall high communication subscale score may be due to the effective skills that parents/caregivers have developed for communicating with healthcare providers.

### Internal consistency reliability

The PedsQL™ 3.0 NMM Thai version showed acceptable values that exceeded the minimum alpha coefficient standard of 0.70 for internal consistency in all subscales of the child self-report except the ‘About Our Family Resources’ subscale. All subscales of the parent proxy-report exceeded the minimum alpha coefficient standard of 0.70 for internal consistency. These findings are in slight contrast to the reliability estimates for the original English language version and the Chinese language translation [2, 5, 7], both of which exceeded 0.70 in all subscales for both the child self-report and the parent proxy-report. One possible explanation for this difference is that in previous studies, the questionnaire was tested in a population with the same disease [2], while our study included subjects with different neuromuscular diseases. The child self-report ‘About Our Family Resources’ subscale found alpha coefficients of less than 0.70. This low internal consistency may be related to the small number of items that compose the subscales, as well as the small sample size [28]. Alpha coefficient values may also be influenced by the low level of schooling within the sample [29]. Total score and other subscales in both the child self-report and the parent proxy-report demonstrated reliability that is acceptable for group comparisons. Subscales that do not exceed the minimum alpha coefficient should only be considered for descriptive analyses.

### Test-retest reliability

In the absence of important changes in clinical status, reliable instruments yield consistent results over repeated administrations to the same individual. A period of 2 to 14 days is considered an adequate interval between tests to reduce the memory effect, and not too lengthy a duration within which clinical symptoms would normally be significantly changed [30].

Test-retest responses demonstrated good to excellent agreement in all subscales for parent-proxy and ‘Total Score’ and ‘About My Neuromuscular Disease’ for child self-reported across a 2–4 weeks’ time period, which suggests that the PedsQL™ 3.0 NMM Thai version is stable and reliable for parent-proxy version and child self-reported version in ‘Total Score’ and ‘About my Neuromuscular disease’; Similar findings in previous studies [2, 5, 7].

However, child self-report in the subscale of ‘Communication’ and ‘Family resources’ showed poor to fair agreement. This finding is different from previous studies [2, 5, 7] that showed moderate to good agreement. The possible explanation could be from the differences in the patient population, in previous studies the researcher studied in more homogenous population with same diseases e.g. DMD or SMA, in our study, we included all children with different neuromuscular disorders that it could affect the changes in the aspects of communication and family resources over time. It can also be concluded that serial measurements are not reliable for serial testing in ‘Communication’ and ‘Family Resources’ in child self-reported.

### Parent-child agreement

There was good to excellent concordance between the perceptions of parents and their children in most subscales except the ‘Communication’ and ‘About Our Family Resources’ subscales which are poor to fair. These findings were similar to those observed from studies in the original English language version [5, 7] and Chinese version [2] of the PedsQL™ 3.0 NMM. Responses to the child self-reports and parent proxy-reports were found to be discordant in many completed HRQoL questionnaires from children with and without chronic illness [31, 32].

### Known-group validity

Known-group validity was assessed using the known-groups method to compare ambulatory and non-ambulatory groups. The measurements were able to discriminate between the two groups. The ‘About My Neuromuscular Disease’ subscale and ‘Total score’ in the child self-report was significantly related to ambulatory status. All subscales and the Total score in the parent proxy-report were also significantly related to ambulatory status. In the child self-report, only the subscales “Communication” and “About Our Family Resources” were unable to discriminate between the two groups. This may be due to the fact that children with chronic diseases most often do not directly communicate with healthcare professionals, and they often do not have clear insight into family situations. The mean total score was 67.86, and the mean score in each subscale in the child self-report was similar to the findings of a US study [7] that administered the questionnaire to spinal muscular atrophy patients. However, compared to a previous study conducted in DMD patients, our results showed higher scores [2, 5]. Differences in patient populations may explain these differences between studies.

With the validated questionnaire, the PedsQL™ 3.0 NMM allows us to follow the changes in health-related quality of life of the children with NMD in the clinic. Knowing the changes in score and the subset of each domain also helps improve patient care.

### Future research

The data from the study is applicable in future researches where it can be applied to disease management research as a tool to monitor the changes of clinical and quality of life outcomes. The future studies to look into psychometric properties of the Thai PedsQL NMM is crucial. Future studies could look into testing of construct validity using either exploratory factor analysis or confirmatory factor analysis [33], to examine the criterion-related validity of PedsQL with other validated questionnaire (administered both Thai PedsQL generic questionnaire [9] and Thai PedsQL NMM in the same (children with NMD) population, and to use other type of testing theory (e.g. Rasch model) to provide other psychometric information for the Thai PedsQL NMM [34].

### Limitations

Our study has some limitations. We used a small convenience sample from a single-center, which could limit the generalizability of our findings. Our institute is the only center in Thailand that provided multi-disciplinary services to children with NMDs, thus the result of this study will be applicable and targeted to pediatric neuromuscular clinic. A larger sample would also have improved the subscale analysis in this population. Finally, we did not evaluate the responsiveness of the instrument to detect shifts in HRQoL as a patient’s health status changes over time, which is a quality that can be regarded as additional evidence of instrument validity [2].

## Conclusions

We conclude that the PedsQL™ 3.0 NMM Thai version has good reliability and validity, and that it is a useful instrument for measuring disease specific health-related quality of life in Thai children with NMD. It can also be used as an outcome measurement in both clinical practice and research. Finally, we suggest further study in a larger NMD population from multiple centres to better assess factor analysis.

## Data Availability

The datasets generated and/or analyzed during the current study are not publicly available to preserve the privacy of the participants but are available from the corresponding author on reasonable request.

## Abbreviations

NMDs: Neuromuscular disorders
PedsQL™ 3.0 NMM: The Pediatric Quality of Life™ 3.0 Neuromuscular Module
HRQoL: Health-related quality of life
ICC: Intraclass correlation coefficient
AHCs: Anterior horn cells
NMJs: Neuromuscular junctions
PedsQL™: The Pediatric Quality of Life
SiRB: Siriraj Institutional Review Board
SPSS: Statistical Package for the Social Sciences
DMD: Duchenne muscular dystrophy
CMT: Charcot-Marie-Tooth
SMA: Spinal Muscular Atrophy
